# Outcomes of urgent coronary artery bypass grafting in patients who have recently recovered from COVID-19 infection, with a median follow-up period of twelve months: our experience

**DOI:** 10.1186/s43044-022-00304-7

**Published:** 2022-09-08

**Authors:** Sudipto Bhattacharya, Ashok Bandyopadhyay, Satyabrata Pahari, Sankha Das, Ashim Kumar Dey

**Affiliations:** 1 Department of Cardiothoracic and Vascular Surgery, Peerless Hospitex Hospital & B K Roy Research Centre, 360, Pancha Sayar Road, Sahid Smrity Colony, Pancha Sayar, Kolkata, West Bengal 700094 India; 2 Department of Anaesthesiology, Peerless Hospitex Hospital & B K Roy Research Centre, 360, Pancha Sayar Road, Sahid Smrity Colony, Pancha Sayar, Kolkata, West Bengal 700094 India

**Keywords:** COVID-19, Coronary artery disease, Coronary artery bypass, Off-pump, Treatment outcomes, Case report

## Abstract

**Background:**

The Coronavirus disease 2019 (COVID-19) was declared a worldwide pandemic in 2020 by the World Health Organization (WHO). Certain individuals are at higher risk, (age > 65 years, pre-existing lung or heart conditions, diabetes and obesity) especially those requiring cardiac surgery, including Coronary Artery Bypass Grafting (CABG). Here we present a case series of 11 patients, operated between April 2020 and April 2022, all of whom had recently recovered from COVID-19, who presented with unstable angina, and therefore required urgent Coronary Artery Bypass Grafting (CABG). Similar cases reported in the past, have had a high morbidity and mortality rate.

**Case presentation:**

The study included 11 males, and their age varied between 53 and 68 years (median of 65 years). They were either partially or fully vaccinated. All of them had a history of recent mild COVID-19 infection. The European system for cardiac operative risk evaluation, EuroSCORE II in-hospital mortality risk at admission, varied between 1.48% and 5.12%. Six out of 11 patients (54.55%) had a recent Acute Coronary Syndrome (ACS) which is associated with a higher risk and poor prognosis. All of them underwent urgent CABG (10 of them, 90.91% cases, using the off-pump technique and one patient had to be converted to the on-pump beating heart surgery technique during surgery). Ten of the 11 patients were operated using the off-pump technique, and there was one death (9.09%). All surviving patients made an uneventful recovery and have been followed up with a median follow-up period of 12 months.

**Conclusions:**

Previous studies on a similar group of patients have resulted in high morbidity and mortality. A conscious effort was made to perform all surgeries off-pump, thereby eliminating the inflammatory effects and other hazards of cardiopulmonary bypass in this case series, with only one out of 11 (9.09%) being converted to the on-pump beating heart technique due to the hemodynamic instability faced during surgery. Our findings show a mortality rate of 9.09%, with the surviving patients doing well at a median follow-up period of 12 months, suggesting that it is a safe procedure in this patient subset.

## Background

The Coronavirus disease 2019 (COVID-19) was declared a worldwide pandemic in 2020 by the World Health Organization (WHO). Globally, there have been 551,226,298 confirmed cases of COVID-19, including 6,345,595 deaths, reported to the WHO [1]. In India, from the 3rd of January 2020 to the 8th of July 2022, there have been 43,585,554 confirmed cases of COVID-19 with 525,343 deaths, reported to WHO. These numbers tell a story regarding the global burden of this disease.

The deleterious effects of COVID-19 infection on the heart and cardiovascular system have been well documented [2]. The Center for Disease Control and Prevention has stated that certain individuals are at higher risk in the setting of the pandemic and should avoid close contact with others. This specifically includes patients aged older than 65 years and those with lung or heart conditions, diabetes, and obesity. This actually represents most of the population who require cardiac surgery [3], including Coronary Artery Bypass Grafting (CABG). A classification system was proposed during the pandemic to categorize and prioritize common cardiac surgery procedures [3, 4] and patients having significant Left Main stem lesions, with unstable angina, were grouped into ‘tier 3’ or ‘high acuity’, where the recommended action was to not defer the surgery, in the form of CABG [4]. We present a case series of 11 patients, who had recently recovered from COVID-19 infection, who underwent urgent CABG, mostly using the off-pump technique (10 out of 11 cases, with one case being converted to an on-pump beating heart procedure, owing to the hemodynamic instability resulting from the positioning of the heart for grafting the Left Anterior Descending artery (LAD), for the sequential anastomosis, using the Left Internal Thoracic Artery (LITA), after having grafted the Diagonal artery. One patient died in hospital, and the rest have survived, are asymptomatic and doing well with a median follow-up period of 12 months.

In each of these 11 cases reported here, a conscious effort was made, to use the Off-Pump technique of coronary artery bypass (OPCAB). There was only one instance where we had to convert to a beating heart on-pump coronary artery bypass (ONCAB) when the heart was not tolerating positioning for the graft to the LAD.

The aim of this study was to study the short-term and the mid-term outcomes of urgent CABG on patients who had recently recovered from COVID-19, to see if COVID-19 vaccination offered an advantage as regards morbidity and mortality and whether the off-pump advantage is applicable to this subset of patients, as regards morbidity and mortality.

### Case presentation

From April 2020 to April 2022, we considered all such patients who had recently recovered from COVID-19 infection, to have undergone surgical coronary revascularization, on an urgent basis, within one month of recovery from COVID-19, to be a part of our study. The primary end point of the study was all-cause mortality during the course of the study, and the secondary end points of the study were myocardial infarction, stroke, infection, sepsis, new renal replacement therapy, need for re-operation and need for repeat revascularization during the course of the study.

### Ethical statement

Written, informed consent was obtained from each patient, for the procedure and further management. Written, informed consent was obtained from each patient, to be a part of this study and for use of clinical data relevant to this study for publication. In case of mortality, the first degree relative of the deceased, gave written, informed consent for use of clinical data relevant to this study for publication.

### Statistical statement

Statistical analysis would be shown in the form of percentages or mean/median for the data subset, as applicable.

A summary of our clinical findings for this case series is summarized in Table 1. The study included 11 male patients, and their age varied between 53 and 68 years (median of 65 years). They were either partially or fully vaccinated (having received one or two doses of the COVID-19 vaccine in India, namely the COVISHIELD™ vaccine (manufactured by Serum Institute of India Pvt. Ltd). Three patients received two doses of the vaccine (27.27%) (and therefore they were fully vaccinated), while the rest were partially vaccinated (each of them received one dose and were awaiting their second dose at admission). All of them had a history of recent mild COVID-19 infection. (Clinical spectrum of the disease is shown in Table 2.) The European system for cardiac operative risk evaluation, EuroSCORE II mortality risk [5] at admission varied between 1.48% and 5.12% for these 11 patients. Six of the 11 patients (54.55%) of this series had a recent onset myocardial infarction or acute coronary syndrome, and were therefore at greater risk. All of them underwent CABG on an urgent basis, sticking to the present recommendations [2, 3] (10 of them, using the off-pump technique and one patient had to be converted to the on-pump beating heart surgery technique during surgery). They received two to four grafts on table, with a median of 3 grafts. One patient, with a previous history of stroke, had a neurological event following surgery (stroke). He was re-intubated, had bradycardia, hypotension, followed by cardiac arrest on the 10th day following surgery and all attempts of resuscitation failed. This death resulted in a mortality rate of 9.09% in our case series. The rest of the patients who survived the procedure, made an uneventful recovery, except atrial fibrillation in one out of 11 patients (9.09%), and they were discharged home on the 7th or the 8th day following surgery and have been doing well at a median follow-up period of 12 months.

**Table 1:** Summarizing our findings in this case series of 11 patients who underwent urgent Coronary Artery Bypass Grafting within a month of recovering from COVID-19 infection

Serial number	Age in years	Sex	COVID-19 Vaccination status	Risk factors	EuroSCORE II mortality risk	Symptoms at presentation	Gap between COVID 19 detection and Surgery	COVID 19 disease severity	CAG findings	Operative details	Post-operative cardiovascular complication	Post-operative respiratory complication	Duration of mechanical ventilation in hours	Post-operative neurological complication	Post-operative renal complication	Duration of hospital stay	Clinical outcome	Duration of follow-up
1	53	Male	Partially vaccinated (received one dose)	HTN, Asthma, Hypothyroidism, recent MI, alcoholism, moderate LV dysfunction, Status-post PCI to LAD and RCA (2017)	3.97%	UA	14 days	Mild	LM DVD with 60–70% LAD stenosis distal to the patent stent	OPCAB, Skeletonized LITA-->LAD, RSVG-->OM	None	None	8	None	None	7 days	Uneventful recovery	18 months
2	65	Male	Partially vaccinated (received one dose)	Elderly, HTN, DM, history of stroke, seronegative polyarthritis	3.28%	UA	14 days	Mild	TVD	OPCAB, Skeletonized LITA--> LAD, RSVG-->RI, There was extensive scarring and akinesia of the inferior wall, densely atheromatous, calcified RCA and thin calibre, non-graftable, PDA and OMs	Bradycardia, hypotension, death	Re intubation	212	Stroke	None	10 days	In-hospital mortality	Not applicable
3	68	Male	Partially vaccinated (received one dose)	HTN, DM, recent MI, moderate LV dysfunction	4.13%	UA	30 days	Mild	TVD	OPCAB, Skeletonized LITA-->LAD, RSVG-->OM, RCA was of uniformly thin calibre, diffusely diseased and non-graftable on table	None	None	8	None	None	7 days	Uneventful recovery	18 months
4	65	Male	Partially vaccinated (received one dose)	HTN, ex-smoker, recent MI	4.13%	UA	26 days	Mild	LM TVD	OPCAB, Skeletonized LITA-->LAD, RSVG-->RI, RSVG--> PDA	None	None	8	None	None	8 days	Uneventful recovery	18 months
5	64	Male	Partially vaccinated (received one dose)	Elderly, HTN, DM, Hypothyroid, recent MI	3.80%	UA	29 days	Mild	TVD	OPCAB, Skeletonized LITA-->LAD, RSVG-->OM, RSVG-->PDA	None	None	8	None	None	8 days	Uneventful recovery	12 months
6	64	Male	Partially vaccinated (received one dose)	HTN, DM, smoker	5.12%	UA	30 days	Mild	TVD with total cut-off of proximal third of LAD (LM equivalent)	OPCAB, Skeletonized LITA-->Diagonal--> LAD(sequential grafts), RSVG-->RI-->OM (sequential grafts), RSVG--> distal RCA	Atrial fibrillation	None	8	None	None	8 days	Uneventful recovery	12 months
7	53	Male	Partially vaccinated (received one dose)	HTN, DM, smoker, dyslipidemia, recent MI	2.44%	UA	28 days	Mild	TVD with ostial stenosis of LAD (LM equivalent)	OPCAB, Skeletonized LITA-->LAD, RSVG-->OM, RSVG-->distal RCA	None	None	8	None	None	8 days	Uneventful recovery	12 months
8	68	Male	Fully vaccinated (received two doses)	HTN, smoker, CKD, recent MI, moderate LV dysfunction	3.93%	UA	28 days	Mild	TVD with 95–100% stenosis in the proximal third of LAD (LM equivalent)	OPCAB, Skeletonized LITA-->LAD, RSVG natural ‘Y’, one limb--> OM, other limb of the ‘Y’--> PDA, one proximal anastomosis on to the ascending thoracic aorta	None	None	6	None	None	8 days	Uneventful recovery	12 months
9	55	Male	Partially vaccinated (received one dose)	HTN, DM, smoker	1.48%	UA	30 days	Mild	TVD	OPCAB, Skeletonized LITA-->LAD, RSVG-->OM, RSVG-->PDA	None	None	7	None	None	8 days	Uneventful recovery	12 months
10	65	Male	Fully vaccinated (received two doses)	HTN, uncontrolled DM, CKD, Dyslipidemia, ex-smoker, had stroke with residual neurodeficit in the right upper limb, moderate LV dysfunction	3.76%	UA	28 days	Mild	TVD	Started as off pump beating heart surgery, with the Diagonal artery being grafted, went on pump (ONCAB), on the beating heart, when there was haemodynmic instability on positioning the heart for grafting the LAD sequentially. Skeletonized LITA-->Diagonal--> LAD(sequential), RSVG--> OM, RSVG--> PDA	None	None	6	None	None	7 days	Uneventful recovery	12 months
11	66	Male	Fully vaccinated (received two doses)	HTN, DM, smoker, alcoholism, Cannabis addiction, strong family history of ischemic heart disease, moderate LV dysfunction	3.91%	UA	24 days	Mild	LM TVD	OPCAB, Skeletonized LITA-->LAD, RSVG--> OM, RSVG--> PLV	None	None	10	None	None	7 days	Uneventful recovery	6 months

*CAG *Coronary angiography, *CKD* Chronic kidney disease, *COVID-19 *Coronavirus disease 2019, *DM *Type 2 diabetes mellitus, *DVD *Double vessel disease, *HTN *Hypertension, *LAD *Left anterior descending artery, *LITA *Left internal thoracic artery, *LM *Significant left main stem lesion, *LV *Left ventricular, *MI* Myocardial infarction, *OM *Obtuse marginal artery, *ONCAB *On pump coronary artery bypass, *OPCAB *Off-pump coronary artery bypass, *PDA *Posterior descending artery, *PLV *Posterior left ventricular artery, *RCA *Right coronary artery, *RI *Ramus intermedius, *TVD *Triple vessel disease, *UA*  Unstable angina

**Table 2:** The present classification for severity of COVID-19 infection. All cases of this series had a history of mild infection

Asymptomatic or Pre-symptomatic Infection: Individuals who test positive for SARS-CoV-2 using a virologic test (i.e., a nucleic acid amplification test [NAAT] or an antigen test) but who have no symptoms that are consistent with COVID-19
Mild Infection: Individuals who have any of the various signs and symptoms of COVID-19 (e.g., fever, cough, sore throat, malaise, headache, muscle pain, nausea, vomiting, diarrhea, loss of taste and smell) but who do not have shortness of breath, dyspnea, or abnormal chest imaging
Moderate Infection*:* Individuals who show evidence of lower respiratory disease during clinical assessment or imaging and who have an oxygen saturation (SpO2) ≥ 94% on room air at sea level
Severe Infection*:* Individuals who have SpO2 < 94% on room air at sea level, a ratio of arterial partial pressure of oxygen to fraction of inspired oxygen (PaO2/FiO2) < 300 mm Hg, a respiratory rate > 30 breaths/min, or lung infiltrates > 50%
Critical Illness: Individuals who have respiratory failure, septic shock, and/or multiple organ dysfunction

Reference: COVID-19 treatment guidelines https://www.covid19treatmentguidelines.nih.gov/ (Accessed on 6/21/2022.)

Each of the discharged patients has been followed up by the cardiac surgical team and the COVID-19 team of physicians at our hospital.

Thus, as regards the primary end point of our study, there was one in-hospital mortality, following stroke, leading to an all-cause mortality of 9.09%. As regards secondary end points of the study, there was no incidence of myocardial infarction, new onset arrhythmia, infection, sepsis, need for new renal replacement therapy, re-operation or need for repeat revascularization. One patient had stroke in the post-operative period (9.09%). One patient had new onset atrial fibrillation (9.09%), which was managed conservatively with medications.

## Discussion

Patients with acute coronary syndrome (ACS) who are infected with COVID-19, often have a poor prognosis. In patients with ACS, cardiac functional reserve may be reduced due to the ongoing myocardial ischemia or necrosis. Therefore, cardiac insufficiency is likely, resulting in sudden clinical deterioration of these patients. Some of the patients with COVID-19 in Wuhan had previous ACS, and they had severe illness and high mortality. For patients with cardiac insufficiency who have underlying heart disease, COVID-19 infection may act as a precipitating factor to worsen the clinical condition and lead to death of the individual [2]. Six of the 11 patients (54.55%) of this case series had a recent onset myocardial infarction or acute coronary syndrome, and were therefore at greater risk.

### On-pump or off-pump is the question

Previous studies have demonstrated a high morbidity and mortality in this group of patients with COVID-19, or those who have recently recovered from the disease and undergoing cardiac surgery [6–10]. There has been one recent study [11] demonstrating an eighteen month follow-up data for a discharged patient, who had COVID-19 and was operated via the Off-Pump technique of Coronary Artery Bypass on the beating heart (OPCAB), where the authors mentioned that avoiding cardiopulmonary bypass (CPB) in such cases could lead to better outcomes, given the systemic inflammatory response, increased damage to red blood cells, damage, platelets, raised catecholamine levels, complement activation, protein denaturation, raised extracellular fluid volumes, stroke and damaging effects on the heart, kidneys, liver, lungs and non-pulsatile flow, hypothermia, duration of CPB, hypoperfusion, and gaseous or particulate microemboli, which produce end-organ injury, all of which may be aggravated in the background of a COVID-19 infection. In Asia, Off-pump Coronary Artery Bypass is more popular with more than 65% of surgical coronary revascularization being performed off-pump in Japan [12] and the numbers are not too different in the rest of Asia. Kowalewski et al. in their meta-analysis showed that high-risk patients had better short-term outcomes when operated via the off-pump technique [13]. Marui et al. [14] showed that OPCAB, as compared to ONCAB, was associated with short-term and long-term benefits in stroke prevention in patients at higher risk as estimated by EuroSCORE. Lemma et al. showed that OPCAB reduces early mortality and morbidity in high-risk patients [15]. Again, in a review of available literature comparing ONCAB and OPCAB [16], Chivasso et al. concluded that the results of the OPCAB technique depended on the surgeon expertise, centre volume and commitment towards this surgical technique and our centre has been doing approximately 90% of our surgical revascularizations using the off-pump technique over the last decade or so, with excellent outcomes. Therefore, there was a conscious effort from our end, to use the Off-Pump technique of coronary artery bypass (OPCAB). There was only one instance where we needed to convert to a beating heart on-pump coronary artery bypass (ONCAB) when the heart was not tolerating positioning for the graft to the LAD. A sequential anastomosis was being done, using the LITA, and the LAD was to be grafted after completing the distal anastomosis to the Diagonal artery. But during positioning of the heart for the graft to the LAD, the patient had hypotension and bradycardia, for which purse strings were taken (on the aorta as well as on the right atrium) and the rest of the anastomoses were completed on the on-pump normothermic beating heart following cannulation of the aorta and the right atrium. Beating heart ONCAB was preferred to conventional ONCAB in this case, to save time and prevent complications like increased blood loss and peri-operative myocardial infarction, as documented in previous studies [17].

One recent study on 12 patients (with an in-hospital mortality rate of 16.67%) suggests that cardiac surgery may be performed safely in a similar subset of patients, especially in the asymptomatic to mild category of COVID-19 infection [18]. Our findings in evaluating outcomes of urgent CABG in patients recently recovered COVID-19 and show a mortality rate of 9.09%, with the surviving patients doing well at a median follow-up period of 12 months, suggesting that it is a safe procedure in this patient subset. But we need more data, especially multi-centre studies, in fully vaccinated, partially vaccinated and unvaccinated individuals, to know better. We also suggest one of the standard worldwide risk scoring systems like the EuroSCORE II and the Society of Thoracic Surgeons Score (STS Score), to incorporate the status of recent past and present COVID-19 infection and the status of vaccination (unvaccinated or partially vaccinated status) to judge the mortality risk in a better manner in the future as COVID-19 and its multiple variants and mutants look like they are here to stay. There being a paucity of follow-up data for this subset of patients, ours has been an effort to bridge this gap.

## Conclusions

Previous studies on a similar group of patients have resulted in high morbidity and mortality. A conscious effort was made here to perform all surgeries off-pump, thereby eliminating the inflammatory effects and other hazards of cardiopulmonary bypass in this series, with only one out of 11 (9.09%) being converted to the on-pump beating heart technique due to the hemodynamic instability faced during surgery. There too, we opted for a beating heart ONCAB technique, rather than the conventional ONCAB technique, to save on time, and prevent complications like increased blood loss and peri-operative myocardial infarction. Our findings show a mortality rate of 9.09%, at a median follow-up period of 12 months, suggesting that it is a safe procedure in this patient subset. But we need more data, especially multi-centre studies, in fully vaccinated, partially vaccinated and unvaccinated individuals, to know better.

## Data Availability

The datasets used and/or analysed during the current study are available from the corresponding author on reasonable request.

## Abbreviations

COVID-19: Coronavirus disease 2019
WHO: World Health Organization
CABG: Coronary artery bypass grafting
OPCAB: Off-pump coronary artery bypass
ONCAB: On-pump coronary artery bypass
LAD: Left anterior descending artery
LITA: Left internal thoracic artery
EuroSCORE II: European system for cardiac operative risk evaluation
ACS: Acute coronary syndrome
STS: Society of thoracic surgeons
